# Spitz Nevus with Features of Clark Nevus, So-Called SPARK Nevus: Case Series Presentation with Emphasis on Cytological and Histological Features

**DOI:** 10.3390/dermatopathology8040055

**Published:** 2021-12-01

**Authors:** Antonietta Cimmino, Gerardo Cazzato, Anna Colagrande, Eugenio Maiorano, Lucia Lospalluti, Giuseppe Ingravallo, Leonardo Resta

**Affiliations:** 1 Section of Pathology, Department of Emergency and Organ Transplantation (DETO), University of Bari “Aldo Moro”, 70124 Bari, Italy; micasucci@inwind.it (A.C.); anna.colagrande@gmail.com (A.C.); eugenio.maiorano@uniba.it (E.M.); giuseppe.ingravallo@uniba.it (G.I.); leonardo.resta@uniba.it (L.R.); 2 Section of Dermatology and Venereology, Department of Biomedical Sciences and Oncology (DIMO), University of Bari “Aldo Moro”, 70124 Bari, Italy; l.lospalluti@gmail.com

**Keywords:** SPARK nevus, dysplastic nevus, Clark, dermatopathology, skin

## Abstract

*Background*: SPARK nevus represents a little-known and characterized entity, with few case series available in the literature. *Methods and results*: we present a case series of 12 patients (6 F and 6 M) between January 2005 and December 2020 and conduct a review of the current literature. Ten articles were selected on the basis of the adopted inclusion criteria and the PRISMA guidelines. *Conclusions*: The definition of histopathological and dermoscopic criteria are important to allow for an agreement to be reached among dermopathologists, and for the development of a consensus on higher case studies. To our knowledge, there are not many case series in the literature, and ours is part of the attempt to increase the knowledge of an entity that remains little-known and characterized.

## 1. Introduction

The Spitz nevus is a benign melanocytic neoplasm that is characterized by two peculiar cytological features: its cells are large, and they are oval (so-called ‘epithelioid’) or spindle in shape [1,2]. On the other hand, Clark nevus is a dysplastic nevus, defined by different criteria, such as the irregularity of melanocytes of junction, the presence of nests that connect the bases of rete ridges (so-called “bridging”), dimension >5 mm, and the eventual presence of scattered melanocytes with large nuclei in the superficial layers of the epidermis [3]. The union of a nevus with cytological features of Spitz nevus with architectural features of Clark’s dysplastic nevus is currently defined as the SPARK nevus. Although this entity was primarily defined “verbally” by B. Ackermann, the first official description in the literature took place only recently, in 2009, on the basis of rather narrow histopathological criteria, such as allowing a correct nosographic diagnosis of these lesions [4]. In this paper we present a case series of lesions found in our routine practice between January 2005 and December 2020. We focus on the histopathological features that cannot be renounced to ‘baptize’ the lesion as “SPARK nevus”, and we conduct a review of the literature on the state of art.

## 2. Materials and Methods

Between January 2005 and December 2020, twelve diagnoses of SPARK nevus were made in as many patients, with all diagnoses confirmed via second opinion at a reference center, as expressed by the latest guidelines of the ESP-EORTC-EURACAN 2021 [5]. All cases were recovered from the archive of our laboratory by means of an electronic file. Sections staining with Hematoxylin/Eosin (EE) and blocks were recovered and re-analyzed by two pathologists experienced in skin pathology (G.C. and A.C.). In the event that there was no agreement, a third dermopathologist (C.A.) was asked. Clinical information was retrieved from fellow dermatologists and plastic surgeons, and when not available, the patient or family members were contacted directly.

In addition, a systematic review was elaborated following the Preferred Reporting Items for Systematic Reviews and Meta-Analyses (PRISMA) guidelines. A search of PubMed and Science.gov databases was performed using the terms: SPARK, Clark Spitzoid in combination with each of the following: Nevus, skin, dermatology. Only articles in English were selected. The last search was run on 21 October 2021. Only manuscripts reporting on patients with histological diagnosis of SPARK Nevus were included. Eligible articles were assessed according to the Oxford Centre for Evidence-Based Medicine 2011 guidelines [6].

Review articles, meta-analyses, observational studies, case reports, survey snapshot studies, letters to the editor, and comments to the letters were all included. Other potentially relevant articles were identified by manually checking the references of the included literature. An independent extraction of articles was performed by two investigators according to the inclusion criteria. Disagreement was resolved by discussion between the two review authors.

## 3. Results

**Figure 1:**
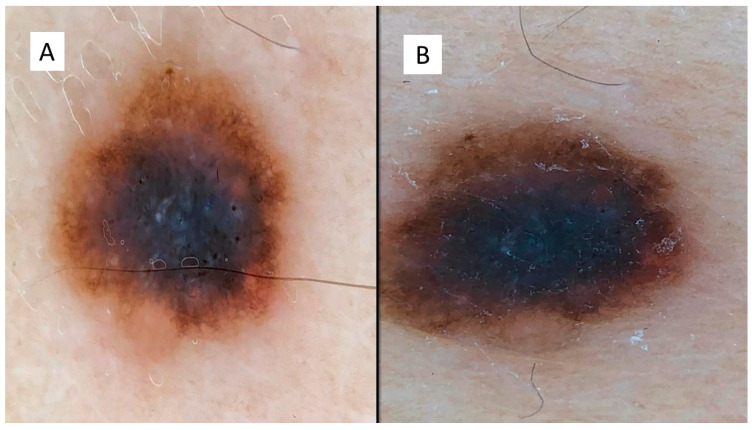
(**A**,**B**) Dermoscopic features of the presented lesions, with a combined pattern in which a homogeneous blackish pattern was present centrally, and in the peripheries, however, there was a regular, bordered lattice.

**Figure 2:**
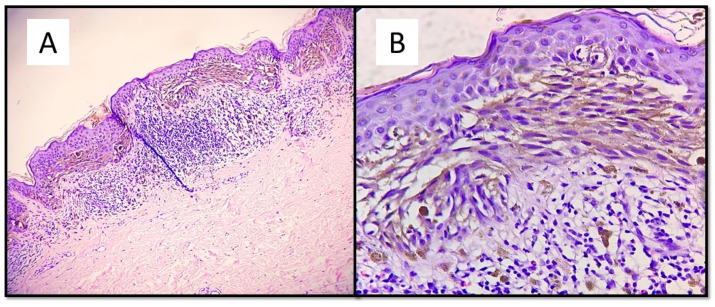

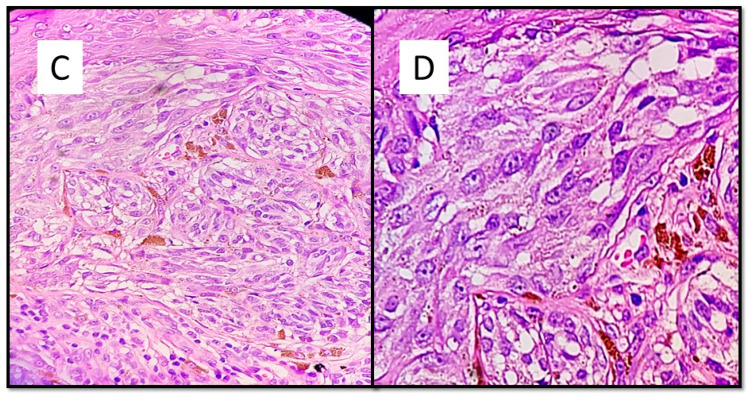
(**A**) This melanocytic proliferation contains features usually found in both Spitz and Clark nevi. Like in. Clark dysplastic nevus, nests are confluent and in parallel with the epidermis. (**B**) Histological details of lentiginous component and some architectural disorder. (Hematoxylin-Eosin, Original Magnification: 4× and 20×). (**C**) As in Spitz nevus, cells are large spindle or epithelioid; moreover, there is an epidermal hyperplasia with ortho-hyperkeratosis, hypergranulosis and acanthosis. (**D**) Citological details of spitzoid cells: nucleus with evident nucleolus and abundant, eosinophilic cytoplasm (Hematoxylin-Eosin, Original Magnification: 10× and 40×.

The case series consisted of 6 females (50%) and 6 males (50%) aged between 12 and 51 years (mean 35.2 years), with more frequent localization in the lower extremities (6 cases, 50%), followed by the back (3 cases, 25%), upper extremities (2 cases, 16.6%) and one head/neck case (8.4%). The diameter of the lesions ranged from a minimum of 3 mm to a maximum of 10 mm, with an average of 5.6 mm, and the time to clinical diagnosis ranged from a minimum of 7 months to a maximum of 18 months, with an average of 10.5 months, excluding 4 cases in which this information was not available. All these data are summarized in Table 1. From the dermoscopic perspective, all the lesions were characterized by a combined pattern in which a homogeneous blackish pattern was centrally present; in the peripheries, however, there was a regular gradient-edged lattice. An example of the lesions is presented in Figure 1A,B. From a histopathological perspective, all the lesions analyzed were made up of spindle-shaped spindle cells, arranged in elongated nests and with a parallel arrangement to the epidermis. This “horizontal” arrangement was reminiscent of Clark’s dysplastic nevus. Furthermore, these melanocytes had an abundant eosinophilic cytoplasm, with a nucleus with thinned chromatin and a central nucleolus (cytological characteristics typical of a Spitz nevus). Sometimes, it was possible to observe a cleft that delimited these melanocytic nests (Figure 2).

At a 2-year follow-up performed on 11/12 patients, there was no recurrence of lesion and/or other local or distant manifestations.

**Figure 3:**
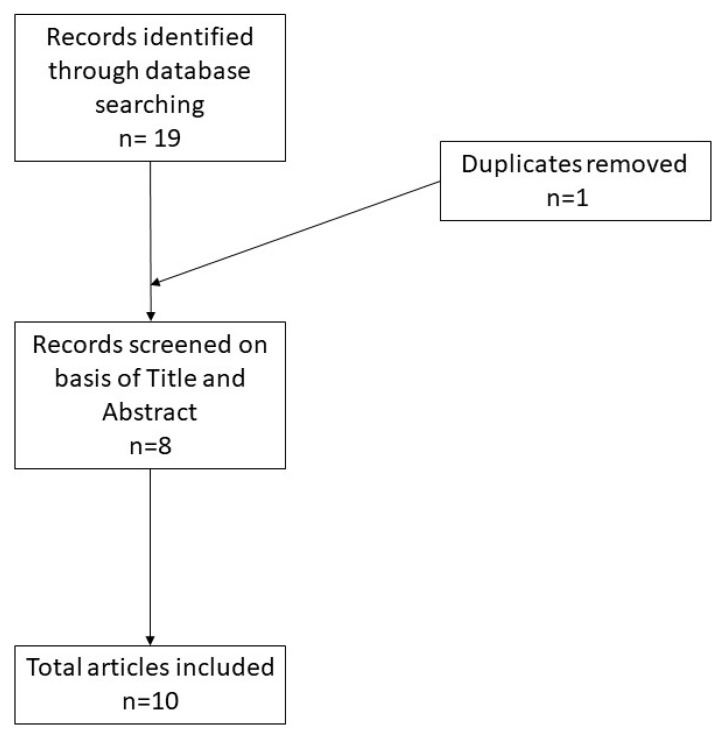
Literature search and article selection.

A total of 19 records were initially identified in the literature search, of which one was a duplicate. After screening for eligibility and inclusion criteria, 10 publications were ultimately included (Figure 3). The majority of publications were reviews (*n* = 5), followed by original articles (*n* = 3) and case reports (*n* = 2). All included studies were rated as level 4 or 5 evidence for clinical research as detailed in the Oxford Centre for Evidence-Based Medicine 2011 guidelines [5].

## 4. Discussion

As noted by Hideko Kamino several years ago, Spitz nevus can have architectural features in common with dysplastic nevi [1,2]. The union of the cytological characteristics of the Spitz nevus (nucleus with evident central nucleolus and abundant cytoplasm, eosinophilic) and the “bridging” architectural pattern of Clark’s dysplastic nevus delineate the extent of the SPARK Nevus, as indicated by other authors such as Spitzoid Clark nevus or “Spastic nevus” (Spitz/dysplastic), “Ditz nevus” (dysplastic/Spitz), or also “dysplastic Spitz” [3,4,5,7,8,9]. The first record of this entity dates to 1999, when Toussaint and Kamino described 67 cases of dysplastic nevi with cytomorphological characteristics of the Spitz nevus [7]. These “dysplastic Spitz nevi” were more common in women and 44% were found in the lower limbs: in our case series, on the other hand, male and female patients were equivalent, with a greater distribution at the level of the lower extremities (more frequently on the thighs). In a study by Barnhill et al. 8 of 95 pigmented spindle cell nevus (PSCN) were termed “PSCN with dysplastic changes”. These showed poor circumscription, lateral extension of the junctional component, elongated rete and lentiginous growth [8].

In another report from 2004, de Giorgi et al. [10] presented a dermoscopic picture of a Spitz nevus combined with a Clark’s nevus and described it as one of the combined nevi. Despite this, it is traditionally believed that the first official description of this entity was made by Glusac et al. in 2009, when twenty-seven nevi with the morphological characteristics of SPARK nevus were presented. In the cited paper, the mean age of the patients was 33 years, and 63% of the patients were female. The lesions were most frequently located in the trunk and lower limbs. Histopathologically, these nevi were composed of large, monomorphic, fused and/or epithelioid melanocytes. The spindle cells were often oriented parallel to the epidermis, with fused mesh and lamellar fibroplasias. Lateral extension of the junctional component was a feature of compound injuries. At a 10-year follow-up of 12/27 patients, there were no relapses and/or metastases [4]. After Fernandez-Flores conducted a careful analysis in 2012 on the eponyms and definitions of less-known nevic lesions [11], in 2018 and 2020 there were authors [12,13] who tried to define the dermoscopic features of the SPARK nevus. Indeed, although the histological criteria allow a fairly certain definition of this entity, greater difficulties exist when trying to understand which dermoscopic characteristics are peculiar to this type of nevus. Biondo et al. [12], reporting their clinical case, hypothesized that the dermoscopic pattern was quite similar to that of Clark’s nevus, while in the case of Bin J. et al. [13], the lesion clinically presented as a brownish asymmetrical patch with a diameter of 1 cm at the periphery and a well-demarcated dome-shaped papule that was centrally located: it could not be clearly distinguished from either the Clark nevus or a pigmented Spitz nevus. From the histopathological point of view, it is important to make a correct differential diagnosis with a form of dysplastic nevus with severe atypia or with a melanoma, and a careful cytological evaluation of the melanocytes present at the dermal–epidermal junction is absolutely mandatory to correctly diagnose this entity without an over- or underdiagnosis [1,14]. Recently, in a paper by Donati et al. the authors report four melanocytic lesions with a MAP2K1 mutation, all showing similar microscopic appearances, including spitzoid cytology and dysplastic architectural features resembling the so-called SPARK nevus, suggesting that these lesions may represent another distinctive group [15].

## 5. Conclusions

The SPARK nevus belongs to one of the least-mentioned types of lesions in the literature. Despite this, it represents an important problem in terms of correct recognition and diagnosis, especially with lesions with a higher degree of malignancy, such as malignant melanoma. The definition of histopathological criteria, but also dermoscopic ones, are important to reach an agreement among dermopathologists and develop a consensus on higher case studies. To our knowledge, there are not many case series in the literature, and ours forms part of the attempt to increase the knowledge of an entity that is still little-known and characterizedy Further studies, with larger case series, are necessary to shed light on a still-unclear nosographic entity.

## Figures and Tables

**Table 1:** Summary of clinical information of our case series.

Number of Patient	Gender	Years	Location	Maximum Diameter	Time to Diagnosis
1	F	41	Back	8 mm	12 months
2	F	26	Right thigh	5 mm	8 months
3	M	12	Left back foot	10 mm	7 months
4	M	36	Right forearm	5 mm	12 months
5	M	35	Right thigh	4 mm	18 months
6	F	51	Left thigh	6 mm	nd
7	F	43	Back	5 mm	nd
8	M	31	Back	6 mm	nd
9	F	28	Left thigh	5 mm	7 months
10	F	40	Left auricle	5 mm	nd
11	M	31	Left leg	9 mm	8 months
12	M	48	Right arm	3 mm	12 months

## Data Availability

Not applicable.
